# HIV vaccine research in Canada

**DOI:** 10.1186/s12981-017-0181-8

**Published:** 2017-09-12

**Authors:** Robin Shattock

**Affiliations:** Department of Medicine, Imperial College London, Norfolk Place, London, W2 1PG UK

I am pleased to have this opportunity to write a preface for this special issue on behalf of the journal “AIDS Research and Therapy”. My research team within Imperial College London (UK) and the European AIDS Vaccine Initiative 2020 (EAVI2020) that I co-ordinate have long-term collaborations with HIV researchers in Canada, and I was invited to attend the Canadian HIV Vaccine Workshop supported by Canadian HIV Vaccine Initiative (CHVI) in Ottawa in December 2016. I was deeply impressed by the success and achievements made by the Canadian HIV vaccine research community. The workshop highlighted the ability of Canadian researchers to make unique contributions in the HIV vaccine research field.

## Current status of HIV vaccine research in the world

HIV vaccine development is one of the greatest biological challenges of our generation. Overcoming viral diversity is a major issue, but many additional challenges will need to be addressed before an effective vaccine can be successfully formulated to achieve the necessary levels of protection required. For example, broadly neutralizing anti-HIV antibodies (bNAbs) that arise during natural HIV-1 infections often take several years to develop and tend to have unusual features which are likely to pose challenges for elicitation through vaccination. These include high levels of somatic mutation, autoreactivity with self-proteins, and long complementarity determining regions [1]. For antibody induction, it is critical that the field better understands the structure of the native Env trimer and the role of the glycan shield in antibody interactions [2–4]. While the induction of bNAbs remains a key goal of HIV research, increasing emphasis is being placed on additional antibody functions beyond classical neutralization including Antibody Dependent Cytotoxicity (ADCC) and phagocytosis (ADCP). While there is much debate over the protective utility of these functions in the absence of neutralization, it is clear they have potential to augment neutralizing activity. Exploiting such mechanisms may enhance the potential efficacy of vaccine candidates. Indeed, two vaccine strategies in late stage efficacy trials are solely dependent on the elicitation of antibodies that only function through FcR mediated mechanisms and display little appreciable neutralization [5]. These studies will prove key in testing whether such functions by themselves are capable of mediating effective protection in individuals at high risk of HIV infection. While development of a prophylactic vaccine remains the primary goal of vaccine research, a renewed interest has been placed on the role of vaccines in effecting long-term remission (effective “cure”) for HIV infected individuals, reducing the need for life-long medication [6]. A desirable long-term goal is to merge parallel B cell and T-cell focused vaccine strategies into an immunological “one-two punch”. This combined approach would incorporate vaccine elements that enable elicitation of antibodies that effectively block infection, coupled with elements that elicit favorable T-cell responses to provide immune-mediated control of breakthrough infections. Without question, the development of a successful HIV vaccine is a sophisticated task and needs collective global effort.

## The current HIV vaccine research in Canada

In the last several decades, Canadian researchers have made significant contribution to HIV vaccine development. Several Canadian researchers have developed their own unique anti-HIV vaccines. For instance, Dr. Yong Kang from UWO, recently tested his whole-killed HIV vaccine (SAV001), in a phase 1 human clinical trial. The vaccine preparation was genetically modified by deleting the nef and vpu genes and fully inactivated by aldrithiol-2 and γ-irradiation [7] while his collaborator, Dr. Yong Gao (UWO) has developed a polyvalent anti-HIV vaccine by utilizing multiple different HIV-1 subtype strains [8]. The Gao lab has found that sequential vaccination strategy could generate broad humoral immune responses (able to neutralize HIV-1 subtype A, B, C and D strains) in a human CD4 B cell transgenic model. Continuing on the theme of invoking a strong humoral response, Drs. Trina Racine, Gary P. Kobinger (UL), and Eric J Arts (UWO) are now working with International AIDS Vaccine Initiative (IAVI) in developing a VSV-based HIV vaccine [9] that will combine unique Canadian research on the HIV-1 Env glycoprotein and on the VSV vaccine vector with the goal of developing a vaccine with a robust and potent anti-HIV immune response with an emphasis on generating quality antibodies to protect against HIV challenge. Again success of any humoral-based vaccine, is dependent of neutralizing antibody production as well as Abs that elicit ADCC. Dr. Andres Finzi (UOM) has been studying how the structural properties of HIV-1 Env might have contributed to the modest efficacy of the RV144 trial and has recently used this knowledge to develop new strategies aimed at sensitizing HIV-1-infected cells to ADCC by easy to elicit non-neutralizing Abs [10].

Segwaying into the role immune cell interactions for optimal anti-HIV responses, Dr. Mario Ostrowski (UOT) has evaluated a number of new adjuvants, such as tumor necrosis factor superfamily (TNFSF) molecules, toll-like receptors (TLRs) agonists, and nucleotide-binding oligomerization domain-containing proteins (NODs) agonists in order to elicit and then maintain a durable and potent memory response from B cells, CD8+ T cells, and NK cells, but avoid overstimulation of HIV-1 susceptible CD4+ T cells [11]. Dr. Dikeakos also attempts to develop HIV-1 nef inhibitors as specific adjuvants [12]. Dr. Ma Luo (UOM) is trying to develop vaccines inducing a T cell response to the highly conserved sequences surrounding the protease cleavage sites (PCS) with the aim of disrupting viral maturation, while limiting excessive immune activation [13]. Variation in epitopes, possible like PCS, could confer resistance but Dr. Michael Grant (MU) has also shown that sequence variants of native immunogenic peptides (termed ‘heteroclitic’ peptides) can generate more robust CD8+ T cell responses and may steer responses away from the phenotypic and functional attributes of exhaustion acquired during chronic HIV infection [14]. As reported by Dr. Larijani [15] sequence changes in epitopes can be induced by APOBEC3G (A3G) and APOBEC3F (A3F)-induced mutations when can effectively diminish CTL recognition. Collectively, these various avenues of vaccine investigation will contribute invaluable and novel information in the design of newer and improved HIV vaccines.

Aside from the preventative vaccines, another important focus to HIV research relates to finding latent HIV in patients receiving cART and how to eradicate this HIV to cure an ongoing infection. Canada has two strong groups of researchers funded by a Gates Foundation-CIHR join intiative. The The Canadian HIV Cure Enterprise (CanCURE) led by Dr. Eric Cohen is focused on finding the elusive latent HIV pool in myeloid cells as well as engaging HIV accessory proteins to find/activate/kill the hidden virus. Dr. Hugo Soudeyns coordinates the Early Pediatric Initiation, Canada Child Cure Cohort Study (EPIC^4^) that is identifying and enrolling HIV infected infants and then providing possible cure strategies. Infants remain an HIV population where a cure may be most feasible. For HIV infected adults and possibly infants receiving effective treatment, Dr. Jamie Mann (UWO) described a new “Kick and Kill” approach to eradicate the established proviral reservoir in HIV patients by utilizing an autologous virus-like-particle therapeutic vaccine [16]. Dr. Kaufmann has set up a novel RNA Flow cytometric fluorescent in situ hybridization (FISH) to detect cytokine mRNA and corresponding protein in single cells for investigations into HIV immunology, vaccination and cure strategies [17].

In parallel to vaccine design efforts other Canadian researchers have been instrumental in studying the mechanisms governing mucosal transmission and susceptibility to infection. Dr. Rupert Kaul (UOT) has provided evidence that the genital microbiome may be an important driver of immune activation in adjacent foreskin tissues while altering this microbiome may be of significant benefit to HIV prevention [18]. To maximize vaccine efficacy, Dr. Jean-Pierre Routy (MGU) proposed that interventions to reduce inflammation might be advantageous at increasing protective HIV vaccine responses by reducing the basal risk of HIV transmission [19]. Innate cellular immunity still remains our first line of defence and Dr. Nicole Bernard has found that an important component of the anti-viral activity of stimulated NK cells that secretion of CC-chemokine (e.g. CCL4) can block HIV entry into new CD4+ target cells [20]. Dr. Keith Fowkes describes a new concept in prevention where instead of targeting the virus, they modulated the host immune system (i.e. mimicking the immune quiescence phenotype) to resist HIV infection [21]. Dr. Kaushic has studied the influence of common mucosal co-factors on HIV infection in the female genital tract, and discussed the role of hormonal contraceptives and bacterial vaginosis on tissue inflammation, T cell immunity and HIV susceptibility [22]. These approaches will be highly valuable for the future development of multi-purpose interventions to prevent HIV transmission and may help to maximize the protective efficacy of novel vaccines.

## Suggestions for further improving HIV vaccine research in Canada

Canadian Federal Government funding towards the development of an HIV vaccine through CHVI (Canadian HIV Vaccine Initiative) has helped to cultivate a Canadian HIV vaccine community with internationally recognized strengths capable of making major contributions. The keen interest shown by IAVI and EAVI to involve Canadian researchers is clear evidence of their ability to make unique contributions. No one country can create a successful vaccine due to prohibitive costs of translational vaccine studies for country-specific funding organizations and the need for centralized facilities, such as transgenic animal models, humanized mouse models, non-human primates, certified good manufacturing practices (GMP), and other expensive infrastructure and resources. Fortunately, these facilities and resources key to vaccine development and testing are available to these multination collaborations largely funded by the Gates Foundation, the US and European funding bodies. It is also critical to maintain a two-pronged approach directing strategic funding towards specific areas where translational progress was most likely, while maintaining support for innovative projects that address unresolved basic and social research issues.

I hope that this special issue will act as a positive signal to government decision makers, indicating that in-roads are being made in HIV vaccine discovery, to the international scientific community that Canadian HIV vaccine research is multifaceted and impactful, to the general public that HIV research is strong in Canada, and more importantly, to communities blighted by HIV, that important scientific advances are being made on multiple fronts. Indeed, as increasing public support is a critical component of advocacy, efforts and accomplishments of the Canadian HIV vaccine research community should be highlighted to a much greater extent at community, funding agency, media and elected government official level.
